# Growth Arrest Triggers Extra-Cell Cycle Regulatory Function in Neurons: Possible Involvement of p27^kip1^ in Membrane Trafficking as Well as Cytoskeletal Regulation

**DOI:** 10.3389/fcell.2019.00064

**Published:** 2019-04-26

**Authors:** Takeshi Kawauchi, Yo-ichi Nabeshima

**Affiliations:** ^1^Laboratory of Molecular Life Science, Institute of Biomedical Research and Innovation, Foundation for Biomedical Research and Innovation at Kobe (FBRI), Kobe, Japan; ^2^Department of Physiology, Keio University School of Medicine, Tokyo, Japan

**Keywords:** growth arrest, p27, neuronal migration, actin cytoskeleton, microtubules, membrane trafficking, golgi apparatus, Rab6

## Abstract

Cell cycle regulation is essential for the development of multicellular organisms, but many cells in adulthood, including neurons, exit from cell cycle. Although cell cycle-related proteins are suppressed after cell cycle exit in general, recent studies have revealed that growth arrest triggers extra-cell cycle regulatory function (EXCERF) in some cell cycle proteins, such as p27(kip1), p57(kip2), anaphase-promoting complex/cyclosome (APC/C), and cyclin E. While p27 is known to control G1 length and cell cycle exit via inhibition of cyclin-dependent kinase (CDK) activities, p27 acquires additional cytoplasmic functions in growth-arrested neurons. Here, we introduce the EXCERFs of p27 in post-mitotic neurons, mainly focusing on its actin and microtubule regulatory functions. We also show that a small amount of p27 is associated with the Golgi apparatus positive for Rab6, p115, and GM130, but not endosomes positive for Rab5, Rab7, Rab8, Rab11, SNX6, or LAMTOR1. p27 is also colocalized with Dcx, a microtubule-associated protein. Based on these results, we discuss here the possible role of p27 in membrane trafficking and microtubule-dependent transport in post-mitotic cortical neurons. Collectively, we propose that growth arrest leads to two different fates in cell cycle proteins; either suppressing their expression or activating their EXCERFs. The latter group of proteins, including p27, play various roles in neuronal migration, morphological changes and axonal transport, whereas the re-activation of the former group of proteins in post-mitotic neurons primes for cell death.

## Introduction

Cell cycle regulation is fundamental for normal development and homeostasis in multicellular organisms. Deregulation of cell cycle causes many severe diseases, such as developmental abnormalities and cancer. Cyclin-dependent kinases (CDKs) and CDK-inhibitory proteins (CKIs) positively and negatively control cell cycle progression as an accelerator and brake, respectively, that make it possible to tightly regulate the cell cycle. Many CDKs, including Cdk1, 2, and 4, are activated via binding to cyclins, whereas Cdk5 is mainly activated by p35 and p39 (Kawauchi, 2014). CKIs are classified into cip/kip and ink4 families. The cip/kip family contains p21^cip1^, p27^kip1^, and p57^kip2^, whereas the ink4 family consists of p16^Ink4a^, p15^Ink4b^, p18^Ink4c^, and p19^Ink4d^ (Sherr and Roberts, 1999; Lu and Hunter, 2010).

Like the other cip/kip proteins, p27^kip1^ (hereafter, p27) binds to a cyclin-CDK complex to suppress the kinase activity of CDKs in general, although p27 does not inhibit the Cyclin D-Cdk4 complex in proliferating cells, where the Cyclin D/Cdk4 enhances the phosphorylation of p27 at Tyr88, resulting in its degradation (Chu et al., 2007; Grimmler et al., 2007; James et al., 2008; Ou et al., 2011). Both cyclin- and CDK-binding domains of p27 are located in the N-terminal. A crystal structure of the N-terminal region of p27, cyclin A and Cdk2 shows that the 3_10_ helix (residues 85–90 in human) of p27 binds deep within the catalytic cleft of Cdk2 and occupies its ATP-binding site (Russo et al., 1996). This structural inhibition of the kinase activity of CDK requires strong binding between p27 and the cyclin-CDK complex, because mutations in the cyclin-binding region of p27 reduce the CDK inhibitory activity of p27 (Vlach et al., 1997).

The protein levels of p27 are high in the G1 phase in proliferating cells. The stability of p27 protein is regulated by its phosphorylation. Phosphorylation at Thr187 or Ser10 promotes or suppresses a proteasome-dependent protein degradation of p27 in S/G2 or G0/G1 phases, respectively (Sheaff et al., 1997; Vlach et al., 1997; Carrano et al., 1999; Montagnoli et al., 1999; Sutterluty et al., 1999; Tsvetkov et al., 1999; Ishida et al., 2000; Malek et al., 2001; Kotake et al., 2005; Kawauchi et al., 2006). While overexpression of wild-type p27 induces G1 arrest in cultured cells, p27 mutated in either cyclin- or CDK-binding region loses the ability to induce G1 arrest, indicating that p27-mediated inhibition of the cyclin-CDK complex suppresses the entry of S phase (Vlach et al., 1997).

In the neural progenitor cells in the developing cerebral cortex, p27 controls the lengths of the G1 phase and cell cycle exit (Mitsuhashi et al., 2001; Tarui et al., 2005). In the postnatal and adult subventricular zones, which provide new neurons that migrate to the olfactory bulb, p27 negatively regulates neurogenesis (Doetsch et al., 2002; Li et al., 2009).

Thus, many studies have indicated that p27 is essential for cell cycle regulation in proliferating cells, including neural progenitors. In addition to its cell cycle-related roles, accumulating evidence indicates that p27 has extra-cell cycle regulatory function (EXCERF) in growth-arrested cells (Kawauchi et al., 2013). Here, we introduce the roles of p27 in cytoskeletal organization and cell migration and discuss its possible involvement in membrane trafficking pathways, with particularly focusing on immature neurons in the developing cerebral cortex.

### EXCERF of Cytoplasmic p27 in Neuronal Migration

p27 is mainly localized in the nucleus to inhibit the activity of cyclin-CDK complexes and this nuclear p27 acts as a tumor suppressor (Blain et al., 2003; Roininen et al., 2019). However, in breast cancer cells, p27 becomes relocalized in the cytoplasm in an Akt-mediated phosphorylation at Thr157-dependent manner, and appears to lose its tumor suppressive activity (Liang et al., 2002; Shin et al., 2002; Viglietto et al., 2002). Interestingly, many cancers, such as ovarian cancer and melanoma, show a correlation between cytoplasmic p27 and malignancy (Rosen et al., 2005; Denicourt et al., 2007). In addition, cytoplasmic p27 promotes the migration of HepG2 hepatocarcinoma cells (McAllister et al., 2003), suggesting that p27 has functionally significant roles outside of the nucleus.

**FIGURE 1:**
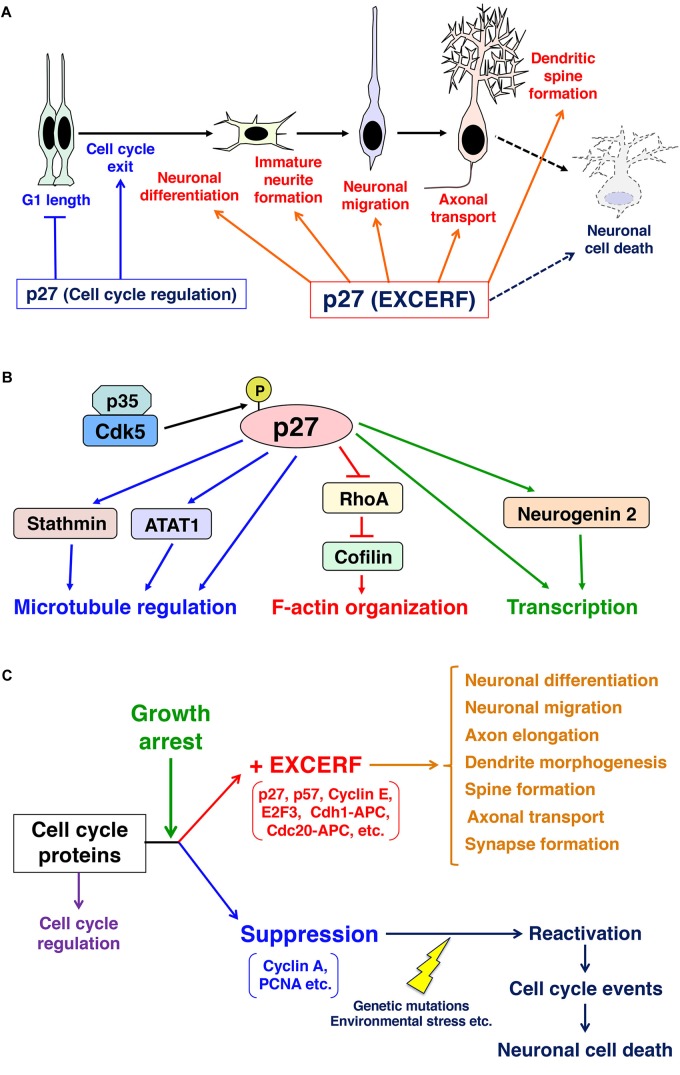
Schematics depicting the roles of p27 in mitotic neural progenitors and post-mitotic neurons. **(A)** p27 controls G1 length and cell cycle exit in neural progenitors. In addition, p27 exhibits many extra-cell cycle regulatory function (EXCERF) in growth-arrested neurons. p27 promotes immature neurite formation, neuronal migration and axonal transport. p27 is also required for dendritic spine maturation and long-term memory in adult hippocampus. **(B)** p27 regulates actin reorganization through the suppression of RhoA and the activation of an actin-binding protein, cofilin. p27 also interacts with microtubules and ATAT1 to control microtubule organization and axonal transport, respectively. **(C)** In post-mitotic cells, cell cycle proteins select two different fates. In general, cell cycle-related proteins, such as cyclin A and PCNA, are suppressed after cell cycle exit and the re-activation of these proteins in growth arrested cells induces cell cycle events, which are priming events to cell death. In contrast, accumulating evidence indicate that other cell cycle-related proteins, including p27, p57, and cyclin E, maintain their expression levels after growth arrest. Growth arrest may switch on extra-cell cycle regulatory functions (EXCERFs) in these proteins.

In primary cortical neurons, p27 is mainly localized in the nucleus but also exhibits punctate localization in the cytoplasm (Kawauchi et al., 2006). *In vivo*, some p27 is localized in the cytoplasm of the immature neurons in the developing cerebral cortex and postnatal subventricular zone (Kawauchi et al., 2006; Li et al., 2009). It has been reported that p27 promotes the migration of immature excitatory and inhibitory neurons in the developing cerebral cortex (Kawauchi et al., 2006; Nguyen et al., 2006; Godin et al., 2012; Nishimura et al., 2014, 2017; Figure 1A). p27 regulates immature neurite formation in multipolar-shaped immature excitatory neurons (Kawauchi et al., 2006) and neurite branching in immature inhibitory neurons (Godin et al., 2012). These effects on cell migration and morphological changes are at least in part dependent on cytoplasmic p27, because p27 is shown to regulate actin and microtubule organization to promote migration (Kawauchi et al., 2006; Godin et al., 2012; Figure 1A). Furthermore, a recent paper shows that p27 controls the acetylation of microtubules via stabilization of α-tubulin acetyltransferase 1 (ATAT1), which modulates axonal transport (Morelli et al., 2018).

### EXCERF of Nuclear p27 in Neuronal Differentiation and Migration

Nuclear p27 also participates in EXCERF, because p27 regulates transcription factors in neurons. p27 interacts with p300 and E2F4 and recruits histone deacetylases and mSIN3A to repress transcription of target genes (Pippa et al., 2012). Furthermore, p27 is shown to regulate gene expression of cell adhesion molecules, including protocadherin-9 and Ncam1 (Bicer et al., 2017). Thus, p27 is associated with various chromatin regions to control transcription.

In the developing cerebral cortex, p27 stabilizes Neurogenin2, a bHLH transcription factor, in cortical neural progenitors and promotes neuronal differentiation (Nguyen et al., 2006; Figure 1A,B). It is also reported that p27 regulates neuronal migration as well as cell cycle exit in cooperation with Rp58, a transcriptional repressor (Clement et al., 2017), but it is unclear whether p27 interacts with Rp58. In the adult hippocampus, neural stem cells give rise to granule neurons of the dentate gyrus throughout life. While p27 is involved in the regulation of stem cell quiescence, this may not result from the EXCERF of p27 (Andreu et al., 2015). The negative regulation of the hippocampal stem cell proliferation depends on the cyclin- and CDK-binding domain of p27. p27 suppresses the kinase activity of Cdk6, which promotes the expansion of hippocampal progenitors (Caron et al., 2018). However, it is unclear whether like in the developing cerebral cortex, p27 also promotes neurogenesis in the adult dentate gyrus through the regulation of transcription.

In differentiating chick retinal ganglion cells (RGCs), p27 is involved in the prevention of extra-DNA synthesis (Ovejero-Benito and Frade, 2015). The differentiating chick RGCs contain tetraploid cells. While the nuclei of some invertebrate neurons, including *Aplysia californica* giant neurons, contain 200,000-fold of the normal amount of haploid DNA, chick RGCs remain tetraploid (or diploid). This may be mediated by the EXCERF of p27, because knockdown of p27 promotes extra-DNA synthesis, which cannot be suppressed by Cdk4/6 inhibition (Ovejero-Benito and Frade, 2015).

### Upstream Factors of p27

As described above, the protein stability of p27 is controlled by its phosphorylation. Cdk5 is an atypical CDK that is activated in post-mitotic neurons in a cyclin-independent manner, whereas Cdk2 binds to cyclin E and controls G1/S transition. Cdk5 is shown to directly phosphorylate p27 at Ser10, which protects it from proteasome-dependent protein degradation (Kawauchi et al., 2006; Figure 1B). In a Cdk5-deficient cerebral cortex, p27 protein levels are reduced in the cytoplasm and nucleus (Zhang et al., 2010), suggesting that Cdk5 regulates the stability of both cytoplasmic and nuclear p27. Ser10 on p27 is also phosphorylated by other kinases, including Dyrk1A and Dyrk1B (Deng et al., 2004; Soppa et al., 2014). Dyrk1A stabilizes p27 and induces cell cycle exit and neuronal differentiation in SH-SY5Y neuroblastoma cells, but the *in vivo* function of this Dyrk1A-mediated regulation of p27 is still unclear.

The Cdk5-p27 pathway plays roles in not only cortical neurons but also non-neuronal cultured cells, including migrating endothelial cells (Li et al., 2006; Liebl et al., 2010). However, p27 can also act upstream of Cdk5 in the cultured neurons treated with Aβ_1–42_ peptide that is a major cause of Alzheimer’s disease. In brains with Alzheimer’s disease, the expression of several cell cycle proteins is abnormally induced (Yang and Herrup, 2007). In response to treatment with Aβ_1–42_ peptide, p27 promotes the formation of a Cdk5-Cyclin D1 complex that dissociates the Cdk5-p35 complex, resulting in neuronal cell death (Jaiswal and Sharma, 2017). It is consistent with previous reports revealing that the induction of Cyclin D1 in post-mitotic neurons leads to cell death (Ino and Chiba, 2001; Koeller et al., 2008), although its underlying mechanism is unclear because Cyclin D cannot activate Cdk5 (Lee et al., 1996). In contrast, the binding of p27 to Cdk5 in the nucleus has been reported to protect neurons from cell death via the suppression of cell cycle events (Zhang et al., 2010). The disruption of the p27 and Cdk5 interaction in nuclei enhances the nuclear export of Cdk5, which deactivates the cell cycle events. Thus, Cdk5 and p27 have multiple functions in neurons and may activate several distinct downstream pathways that are associated with neuronal cell death.

Unlike Cdk5, Cdk2 phosphorylates p27 at Thr187 and the Thr187-phosphorylated p27 binds to Skp2, an E3 ubiquitin ligase, resulting in its degradation (Sheaff et al., 1997; Vlach et al., 1997; Carrano et al., 1999; Montagnoli et al., 1999; Sutterluty et al., 1999; Tsvetkov et al., 1999; Malek et al., 2001). Thus, Cdk5 and Cdk2 exert opposite effects on the protein stability of p27 through phosphorylation at distinct sites. Although Thr187 phosphorylation of p27 is observed in cortical neurons (Kawauchi et al., 2006), it is unclear which kinase(s) contributes to this phosphorylation in post-mitotic neurons, where Cdk2 activity is low.

In addition to these kinases, it has been reported that connexin-43 (Cx43), a component of the gap junction, acts as an upstream regulator of p27. Knockdown of Cx43 reduces the protein levels of p27 in cortical neurons and disturbs the formation of immature neurites in cortical migrating neurons (Liu et al., 2012). Consistently, suppression of Cx43 expression perturbs the neuronal positioning in the developing cerebral cortex (Elias et al., 2007; Qi et al., 2016). However, it is unclear whether this regulation is mediated by Cdk5 or not.

### Downstream Factors of p27: Regulation of Cytoskeletal Organization

What are the underlying mechanisms of the EXCERF of p27? Accumulating evidence indicates that a major downstream pathway targeted by p27 in EXCERF is cytoskeletal regulation. It has been reported that p27 promotes cofilin-mediated actin reorganization in neurons (Kawauchi et al., 2006; Figure 1B). Cofilin severs actin filaments and enhances the depolymerization of actin filaments (Moriyama and Yahara, 1999). Cofilin directly binds to actin filaments but the phosphorylation at Ser3 by LIM kinase decreases its actin-binding affinity (Moriyama et al., 1996; Arber et al., 1998; Yang et al., 1998). LIM kinase is activated by RhoA-Rho kinase/ROCK and Rac1-PAK1 pathways. p27 negatively regulates Ser3-phosphorylation of cofilin via the suppression of RhoA, rather than Rac1, in cortical immature neurons, resulting in the activation of cofilin (Kawauchi et al., 2006). p27 can directly bind to RhoA to inhibit the interaction between RhoA and its activators in non-neuronal cells (Besson et al., 2004). However, the binding affinity of p27 for RhoA is low (Phillips et al., 2018), implying that some upstream signals strengthen the binding of these proteins or that p27 indirectly suppresses RhoA activity in neurons. Interestingly, RSK1 is reported to phosphorylate Thr198 of p27, resulting in enhanced binding between p27 and RhoA (Larrea et al., 2009).

The p27-RhoA-cofilin pathway is important for not only neuronal migration and morphological changes in the developing cerebral cortex but also the establishment of long-term memory in the adult brain. Cks1 knockout mice have increased p27 protein levels and decreased Ser3-phosphorylation of cofilin (that is, cofilin activity is abnormally increased), and impairment of learning and long-term memory (Kukalev et al., 2017). Cks1 is strongly expressed in the hippocampus and required for late-phase long-term potentiation (late LTP) and proper maturation of dendritic spines.

A recent report indicates that p27 binds to another actin-binding protein, Cortactin, in non-neuronal cells (Jeannot et al., 2017). p27 promotes the interaction between Cortactin and PAK1, and PAK1-mediated phosphorylation of Cortactin enhances the turnover of invadopodia. Thus, it is possible that in addition to cofilin, p27 may also regulate the actin-binding protein(s) in cortical neurons.

In the inhibitory neurons in the developing cerebral cortex, p27 interacts with microtubules and promotes its polymerization (Godin et al., 2012). Furthermore, p27 binds to ATAT1 to increase the acetylation of microtubules, as described above (Morelli et al., 2018). Thus, p27 regulates multiple downstream events to control both actin and microtubule cytoskeletal organization (Figure 1B).

### Downstream Factors of p27: Possible Involvement in Membrane Trafficking

**FIGURE 2:**
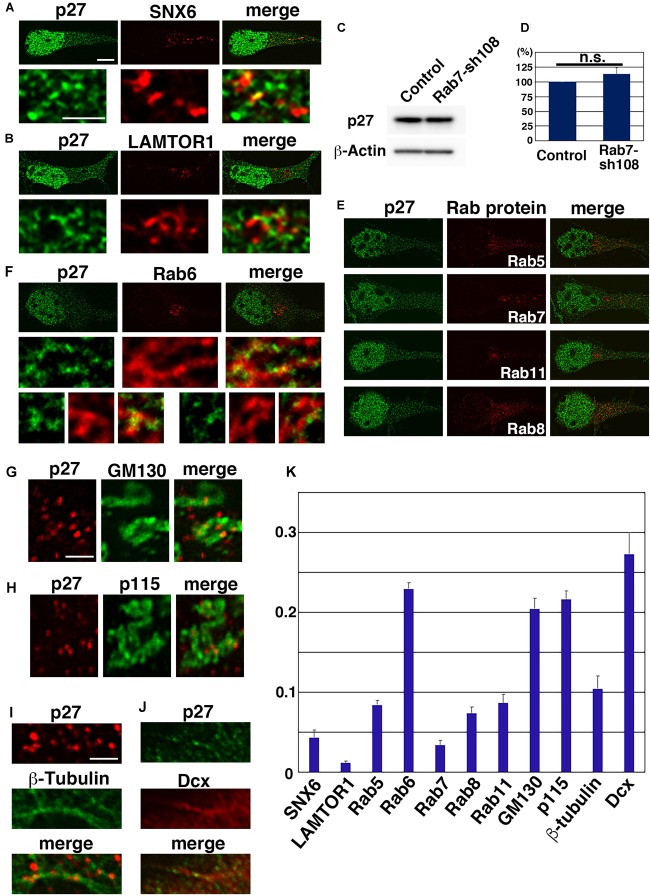
Subcellular localization of p27. **(A,B,E–K)** Primary cortical neurons from E15 cerebral cortices incubated for 2 days *in vitro* and stained with the indicated antibodies. Immunocytochemical analyses were performed as described previously (Shikanai et al., 2018a). Fluorescence images were obtained by A1R laser scanning confocal microscopy with a high sensitivity GaAsP detector (Nikon) using the narrow pinhole size (0.3) and subjected to deconvolution processing with the Richardson-Lucy algorithm in NIS-ER software (Nikon). The graph in **(K)** shows the colocalization efficient (Pearson’s correlation) of the indicated proteins with p27, as determined using NIS elements software (Nikon). Significance was determined by Kruskal-Wallis test with post hoc Steel-Dwass test [< the critical value at 1% (Rab6 vs. SNX6 or LAMTOR1 or Rab5 or Rab7 or Rab8 or Rab11 or β-tubulin; p115 vs. SNX6 or LAMTOR1 or Rab5 or Rab7 or Rab8 or Rab11 or β-tubulin; GM130 vs. SNX6 or LAMTOR1 or Rab5 or Rab7 or Rab8 or Rab11 or β-tubulin. Dcx vs. SNX6 or LAMTOR1 or Rab5 or Rab7 or Rab8 or Rab11 or β-tubulin; Rab5 vs. LAMTOR1 or Rab7; LAMTOR1 vs. Rab8 or Rab11 or β-tubulin)]. **(C,D)** Primary cortical neurons from E15 cerebral cortices transfected with control vector or shRNA-expressing vector targeting for Rab7 (Rab7-sh108 (Kawauchi et al., 2010)) plus pCAG-EGFP (Kawauchi et al., 2003) and incubated for 2 days *in vitro*. Immunoblot analyses were performed as described previously (Kawauchi et al., 2006). The graph in **(D)** shows the ratios of immunoblot band intensities of p27/β-actin ± s.e.m. (*n* = 6). No significant differences (n.s.) between control and Rab7-sh108-transfected neurons were found by Student’s *t*-test (*P* = 0.3232). Primary antibodies used in this figure were anti-p27 (610241, BD Biosciences) (green in **A,B,E,F,J**, and immunoblots in **C,D**), anti-p27 (3686, Cell Signaling Technology) (red in **G–I**), anti-SNX6 (PA5-61948, Thermo Fisher Scientific), anti-LAMTOR1 (8975, Cell Signaling Technology), anti-Rab5 (3547, Cell Signaling Technology), anti-Rab6 (9625, Cell Signaling Technology), anti-Rab7 (9367, Cell Signaling Technology), anti-Rab8 (6975, Cell Signaling Technology), anti-Rab11 (5589, Cell Signaling Technology), anti-GM130 (610822, BD Biosciences), anti-p115 (612260, BD Biosciences), anti-β-tubulin (T5201, Sigma), anti-Dcx (4604, Cell Signaling Technology) and anti-β-actin (A5441, Sigma) antibodies. Scale bars: 4 μm in (**E** and upper panels in **A,B,F**), 1 μm in (lower panels in **A,B**, middle and lower panels in **F**), 1 μm in **(G–J)**.

A major EXCERF of p27 is cytoskeletal organization. In addition to this, several reports suggest roles for p27 in membrane trafficking. In cultured cell lines, including NIH-3T3 and HeLa cells, p27 is colocalized with SNX6, a sorting nexin family protein that controls retrograde vesicular transport from early endosomes to *trans*-Golgi networks (TGNs), and LAMP2, a marker for lysosomes (Fuster et al., 2010). Although p27 is generally degraded in proteasomes, a small fraction of p27 may undergo lysosomal degradation when cells reenter the cell cycle in response to serum stimulation (Fuster et al., 2010). Furthermore, p27 is reported to interact with p27RF-Rho/p18/LAMTOR1 (hereafter, LAMTOR1), which is localized in late endosomes and lysosomes (Hoshino et al., 2009; Hoshino et al., 2011; Takahashi et al., 2012). However, our high-resolution microscopy analyses revealed no colocalization of p27 with SNX6 or LAMTOR1 in primary cortical neurons (Figure 2A,B,K), although some localization of p27 and LAMTOR1 is observed together in the same vesicular components (Figure 2B). In addition, inhibition of lysosomal degradation pathways by knockdown of Rab7 did not significantly affect the protein levels of p27 in cortical neurons (Figure 2C,D).

Given that the degradation of p27 is not dependent on lysosomes, we examined the possible association of p27 with other endosomal pathways. Rab5, Rab7, and Rab11, markers for early, late and recycling endosomes, respectively, are known to regulate cortical neuronal migration, similar to p27 (Kawauchi et al., 2010; Kawauchi, 2012). However, we observed little to no colocalization between p27 and these Rab proteins in primary cortical neurons (Figure 2E,K). A similar result was found with Rab8, a regulator of secretion pathways (Henry and Sheff, 2008; Shikanai et al., 2018b; Figure 2E,K), indicating low association of p27 with endosomal and Rab8-dependent pathways. In contrast, a small percentage of Rab6, a marker for Golgi, seems to be associated with p27. High-resolution microscopy analyses revealed that some p27 associates with Rab6-positive compartments (Figure 2F,K). In addition, a small percentage of p27 is observed at the tubular compartments positive for GM130 or p115, markers for Golgi apparatus (Figure 2G,H,K). These data suggest that p27 may preferentially associate with Golgi apparatus in cortical neurons.

It is unclear whether p27 is associated with the Golgi membrane or not. Considering that p27 binds to microtubules and its associated proteins, stathmin and ATAT1, and regulates axonal transports (Baldassarre et al., 2005; Godin et al., 2012; Morelli et al., 2018), it is possible that p27 regulates microtubule-associated motor proteins to control the intracellular transport of the Golgi and other endosomes/organelles. In fact, some p27-positive puncta were observed along the microtubules (Figure 2I). Furthermore, p27 partially colocalizes with Dcx, a microtubule-regulatory protein that is associated with human X-linked lissencephaly (Figure 2J,K).

Interestingly, knockout of p27 enhances the trafficking of CTxB, a marker for GM1 ganglioside-positive lipid rafts, in cultured fibroblasts possibly due to altered stathmin-mediated regulation of microtubule stability (Belletti et al., 2010), suggesting that p27 negatively regulates lipid raft-mediated endocytosis. In cortical neurons, CTxB is internalized via caveolin-1-mediated endocytosis at least in part (Shikanai et al., 2018a). In addition, a recent report indicates that caveolin-1 enhances the elimination of the immature neurites in cortical neurons (Shikanai et al., 2018a), which is opposite to the effect of p27 that promotes immature neurite formation (Kawauchi et al., 2006). Thus, the observation of p27-mediated microtubule regulation in lipid raft trafficking in non-neuronal cells may be consistent with *in vivo* function of p27 and caveolin-1 in immature neurons in the developing cerebral cortex.

## Conclusion and Future Direction

In this paper, we introduce the EXCERF of p27, such as cytoskeletal organization and membrane trafficking. Other cell cycle-related proteins also exhibit EXCERF (Frank and Tsai, 2009; Kawauchi et al., 2013). For example, p21 and p57 are known to regulate neurite extension and neuronal migration, respectively (Tanaka et al., 2002; Itoh et al., 2007). E2F3, a transcription factor that is negatively controlled by Rb, is also reported to control neuronal migration (McClellan et al., 2007). Cdh1-Anaphase promoting complex (APC) and Cdc20-APC, both of which are E3 ubiquitin ligases, regulate axonal growth and dendrite morphogenesis (Konishi et al., 2004; Kim et al., 2009). Furthermore, cyclin E regulates synapse number and synaptic plasticity through the restraining of Cdk5 activity (Odajima et al., 2011). Thus, many cell cycle-related proteins have functions in G0-arrested neurons (Figure 1C).

Alternatively, expression of other cell cycle-related proteins is suppressed during cell cycle exit. Re-expression of these cell cycle-related proteins, including cyclin A and PCNA, activates cell cycle events in post-mitotic neurons, and leads to cell death and neurodegenerative diseases (Yang and Herrup, 2007; Figure 1C). Consistently, knockdown of p27 in post-mitotic supporting cells in postnatal cochleae induces cell cycle re-entry, but these cells eventually undergo apoptosis (Ono et al., 2009).

Thus, we could classify cell cycle-related proteins into two categories (Figure 1C). One set exhibits EXCERF even in G0-arrested cells, such as post-mitotic neurons. The other set is normally suppressed soon after growth arrest and their re-activation is a trigger to cell death. Growth arrest may be an important signal that activates the EXCERF of the former group of proteins, including p27, p57, APC, and cyclin E, and silences proteins in the latter group. This concept raises many questions to be solved. What happens at the timing of growth arrest? What are the differences between cell cycle proteins with or without EXCERF? What are the global picture and regulatory mechanisms of EXCERF? More specifically, what are the physiological roles of the puncta-like cytoplasmic p27? Future studies will answer these fundamental questions and will consolidate this new concept of EXCERF in cell biology and neuroscience.

## Ethics Statement

For primary culture experiments of mouse embryonic cortical neurons, pregnant ICR mice were purchased from SLC Japan or Animal Facility of RIKEN-BDR. Animals were handled in accordance with guidelines established by RIKEN-BDR and Institute of Biomedical Research and Innovation, FBRI. There is no data using human subjects in this manuscript.

## Author Contributions

TK conceived the project, performed experiments and wrote the manuscript. YN administrated the experimental environments and provided helpful comments.

## Conflict of Interest Statement

The authors declare that the research was conducted in the absence of any commercial or financial relationships that could be construed as a potential conflict of interest.

## References

[B1] Andreu Z. Khan M. A. Gonzalez-Gomez P. Negueruela S. Hortiguela R. San Emeterio J. (2015). The cyclin-dependent kinase inhibitor p27 kip1 regulates radial stem cell quiescence and neurogenesis in the adult hippocampus. *Stem Cells* 33 219–229. 10.1002/stem.1832 25185890

[B2] Arber S. Barbayannis F. A. Hanser H. Schneider C. Stanyon C. A. Bernard O. (1998). Regulation of actin dynamics through phosphorylation of cofilin by LIM-kinase. *Nature* 393 805–809. 10.1038/31729 9655397

[B3] Baldassarre G. Belletti B. Nicoloso M. S. Schiappacassi M. Vecchione A. Spessotto P. (2005). p27(Kip1)-stathmin interaction influences sarcoma cell migration and invasion. *Cancer Cell* 7 51–63. 10.1016/j.ccr.2004.11.025 15652749

[B4] Belletti B. Pellizzari I. Berton S. Fabris L. Wolf K. Lovat F. (2010). p27kip1 controls cell morphology and motility by regulating microtubule-dependent lipid raft recycling. *Mol. Cell. Biol.* 30 2229–2240. 10.1128/MCB.00723-09 20194624 PMC2863592

[B5] Besson A. Gurian-West M. Schmidt A. Hall A. Roberts J. M. (2004). p27Kip1 modulates cell migration through the regulation of RhoA activation. *Genes Dev.* 18 862–876. 10.1101/gad.1185504 15078817 PMC395846

[B6] Bicer A. Orlando S. Islam A. Gallastegui E. Besson A. Aligue R. (2017). ChIP-Seq analysis identifies p27(Kip1)-target genes involved in cell adhesion and cell signalling in mouse embryonic fibroblasts. *PLoS One* 12:e0187891. 10.1371/journal.pone.0187891 29155860 PMC5695801

[B7] Blain S. W. Scher H. I. Cordon-Cardo C. Koff A. (2003). p27 as a target for cancer therapeutics. *Cancer Cell* 3 111–115. 10.1016/s1535-6108(03)00026-6 12620406

[B8] Caron N. Genin E. C. Marlier Q. Verteneuil S. Beukelaers P. Morel L. (2018). Proliferation of hippocampal progenitors relies on p27-dependent regulation of Cdk6 kinase activity. *Cell Mol. Life. Sci.* 75 3817–3827. 10.1007/s00018-018-2832-x 29728713 PMC11105564

[B9] Carrano A. C. Eytan E. Hershko A. Pagano M. (1999). SKP2 is required for ubiquitin-mediated degradation of the CDK inhibitor p27. *Nat. Cell Biol.* 1 193–199. 10.1038/12013 10559916

[B10] Chu I. Sun J. Arnaout A. Kahn H. Hanna W. Narod S. (2007). p27 phosphorylation by Src regulates inhibition of cyclin E-Cdk2. *Cell* 128 281–294. 10.1016/j.cell.2006.11.049 17254967 PMC1961623

[B11] Clement O. Hemming I. A. Gladwyn-Ng I. E. Qu Z. Li S. S. Piper M. (2017). Rp58 and p27(kip1) coordinate cell cycle exit and neuronal migration within the embryonic mouse cerebral cortex. *Neural Dev.* 12:8. 10.1186/s13064-017-0084-3 28506232 PMC5433244

[B12] Deng X. Mercer S. E. Shah S. Ewton D. Z. Friedman E. (2004). The cyclin-dependent kinase inhibitor p27Kip1 is stabilized in G(0) by Mirk/dyrk1B kinase. *J. Biol. Chem.* 279 22498–22504. 10.1074/jbc.m400479200 15010468

[B13] Denicourt C. Saenz C. C. Datnow B. Cui X. S. Dowdy S. F. (2007). Relocalized p27Kip1 tumor suppressor functions as a cytoplasmic metastatic oncogene in melanoma. *Cancer Res.* 67 9238–9243. 10.1158/0008-5472.can-07-1375 17909030

[B14] Doetsch F. Verdugo J. M. Caille I. Alvarez-Buylla A. Chao M. V. Casaccia-Bonnefil P. (2002). Lack of the cell-cycle inhibitor p27Kip1 results in selective increase of transit-amplifying cells for adult neurogenesis. *J. Neurosci.* 22 2255–2264. 10.1523/jneurosci.22-06-02255.2002 11896165 PMC6758265

[B15] Elias L. A. Wang D. D. Kriegstein A. R. (2007). Gap junction adhesion is necessary for radial migration in the neocortex. *Nature* 448 901–907. 10.1038/nature06063 17713529

[B16] Frank C. L. Tsai L. H. (2009). Alternative functions of core cell cycle regulators in neuronal migration, neuronal maturation, and synaptic plasticity. *Neuron* 62 312–326. 10.1016/j.neuron.2009.03.029 19447088 PMC2757047

[B17] Fuster J. J. Gonzalez J. M. Edo M. D. Viana R. Boya P. Cervera J. (2010). Tumor suppressor p27(Kip1) undergoes endolysosomal degradation through its interaction with sorting nexin 6. *FASEB J.* 24 2998–3009. 10.1096/fj.09-138255 20228253

[B18] Godin J. D. Thomas N. Laguesse S. Malinouskaya L. Close P. Malaise O. (2012). p27(Kip1) is a microtubule-associated protein that promotes microtubule polymerization during neuron migration. *Dev. Cell* 23 729–744. 10.1016/j.devcel.2012.08.006 23022035

[B19] Grimmler M. Wang Y. Mund T. Cilensek Z. Keidel E. M. Waddell M. B. (2007). Cdk-inhibitory activity and stability of p27Kip1 are directly regulated by oncogenic tyrosine kinases. *Cell* 128 269–280. 10.1016/j.cell.2006.11.047 17254966

[B20] Henry L. Sheff D. R. (2008). Rab8 regulates basolateral secretory, but not recycling, traffic at the recycling endosome. *Mol. Biol. Cell* 19 2059–2068. 10.1091/mbc.E07-09-0902 18287531 PMC2366880

[B21] Hoshino D. Koshikawa N. Seiki M. (2011). A p27(kip1)-binding protein, p27RF-Rho, promotes cancer metastasis via activation of RhoA and RhoC. *J. Biol. Chem.* 286 3139–3148. 10.1074/jbc.M110.159715 21087931 PMC3024806

[B22] Hoshino D. Tomari T. Nagano M. Koshikawa N. Seiki M. (2009). A novel protein associated with membrane-type 1 matrix metalloproteinase binds p27(kip1) and regulates RhoA activation, actin remodeling, and matrigel invasion. *J. Biol. Chem.* 284 27315–27326. 10.1074/jbc.M109.041400 19654316 PMC2785659

[B23] Ino H. Chiba T. (2001). Cyclin-dependent kinase 4 and cyclin D1 are required for excitotoxin-induced neuronal cell death in vivo. *J. Neurosci.* 21 6086–6094. 10.1523/jneurosci.21-16-06086.2001 11487632 PMC6763149

[B24] Ishida N. Kitagawa M. Hatakeyama S. Nakayama K. (2000). Phosphorylation at serine 10, a major phosphorylation site of p27(Kip1), increases its protein stability. *J. Biol. Chem.* 275 25146–25154. 10.1074/jbc.m001144200 10831586

[B25] Itoh Y. Masuyama N. Nakayama K. Nakayama K. I. Gotoh Y. (2007). The cyclin-dependent kinase inhibitors p57 and p27 regulate neuronal migration in the developing mouse neocortex. *J. Biol. Chem.* 282 390–396. 10.1074/jbc.m609944200 17092932

[B26] Jaiswal S. Sharma P. (2017). Role and regulation of p27 in neuronal apoptosis. *J. Neurochem.* 140 576–588. 10.1111/jnc.13918 27926980

[B27] James M. K. Ray A. Leznova D. Blain S. W. (2008). Differential modification of p27Kip1 controls its cyclin D-cdk4 inhibitory activity. *Mol. Cell. Biol.* 28 498–510. 10.1128/mcb.02171-06 17908796 PMC2223302

[B28] Jeannot P. Nowosad A. Perchey R. T. Callot C. Bennana E. Katsube T. (2017). p27(Kip1) promotes invadopodia turnover and invasion through the regulation of the PAK1/Cortactin pathway. *eLife* 6:e22207. 10.7554/eLife.22207 28287395 PMC5388532

[B29] Kawauchi T. (2012). Cell adhesion and its endocytic regulation in cell migration during neural development and cancer metastasis. *Int. J. Mol. Sci.* 13 4564–4590. 10.3390/ijms13044564 22605996 PMC3344232

[B30] Kawauchi T. (2014). Cdk5 regulates multiple cellular events in neural development, function and disease. *Dev. Growth Differ.* 56 335–348. 10.1111/dgd.12138 24844647

[B31] Kawauchi T. Chihama K. Nabeshima Y. Hoshino M. (2003). The in vivo roles of STEF/Tiam1, Rac1 and JNK in cortical neuronal migration. *EMBO J.* 22 4190–4201. 10.1093/emboj/cdg413 12912917 PMC175802

[B32] Kawauchi T. Chihama K. Nabeshima Y. Hoshino M. (2006). Cdk5 phosphorylates and stabilizes p27kip1 contributing to actin organization and cortical neuronal migration. *Nat. Cell Biol.* 8 17–26. 10.1038/ncb1338 16341208

[B33] Kawauchi T. Sekine K. Shikanai M. Chihama K. Tomita K. Kubo K. (2010). Rab GTPases-dependent endocytic pathways regulate neuronal migration and maturation through N-cadherin trafficking. *Neuron* 67 588–602. 10.1016/j.neuron.2010.07.007 20797536

[B34] Kawauchi T. Shikanai M. Kosodo Y. (2013). Extra-cell cycle regulatory functions of cyclin-dependent kinases (CDK) and CDK inhibitor proteins contribute to brain development and neurological disorders. *Genes Cells* 18 176–194. 10.1111/gtc.12029 23294285 PMC3594971

[B35] Kim A. H. Puram S. V. Bilimoria P. M. Ikeuchi Y. Keough S. Wong M. (2009). A centrosomal Cdc20-APC pathway controls dendrite morphogenesis in postmitotic neurons. *Cell* 136 322–336. 10.1016/j.cell.2008.11.050 19167333 PMC2707082

[B36] Koeller H. B. Ross M. E. Glickstein S. B. (2008). Cyclin D1 in excitatory neurons of the adult brain enhances kainate-induced neurotoxicity. *Neurobiol. Dis.* 31 230–241. 10.1016/j.nbd.2008.04.010 18585919 PMC2614463

[B37] Konishi Y. Stegmuller J. Matsuda T. Bonni S. Bonni A. (2004). Cdh1-APC controls axonal growth and patterning in the mammalian brain. *Science* 303 1026–1030. 10.1126/science.1093712 14716021

[B38] Kotake Y. Nakayama K. Ishida N. Nakayama K. I. (2005). Role of serine 10 phosphorylation in p27 stabilization revealed by analysis of p27 knock-in mice harboring a serine 10 mutation. *J. Biol. Chem.* 280 1095–1102. 10.1074/jbc.m406117200 15528185

[B39] Kukalev A. Ng Y. M. Ju L. Saidi A. Lane S. Mondragon A. (2017). Deficiency of Cks1 leads to learning and long-term memory defects and p27 dependent formation of neuronal cofilin aggregates. *Cereb. Cortex* 27 11–23. 10.1093/cercor/bhw354 28365778 PMC5939225

[B40] Larrea M. D. Hong F. Wander S. A. da Silva T. G. Helfman D. Lannigan D. (2009). RSK1 drives p27Kip1 phosphorylation at T198 to promote RhoA inhibition and increase cell motility. *Proc. Natl. Acad. Sci. U.S.A.* 106 9268–9273. 10.1073/pnas.0805057106 19470470 PMC2695095

[B41] Lee M. H. Nikolic M. Baptista C. A. Lai E. Tsai L. H. Massague J. (1996). The brain-specific activator p35 allows Cdk5 to escape inhibition by p27Kip1 in neurons. *Proc. Natl. Acad. Sci. U.S.A.* 93 3259–3263. 10.1073/pnas.93.8.3259 8622924 PMC39593

[B42] Li X. Tang X. Jablonska B. Aguirre A. Gallo V. Luskin M. B. (2009). p27(KIP1) regulates neurogenesis in the rostral migratory stream and olfactory bulb of the postnatal mouse. *J. Neurosci.* 29 2902–2914. 10.1523/JNEUROSCI.4051-08.2009 19261886 PMC3488282

[B43] Li Z. Jiao X. Wang C. Ju X. Lu Y. Yuan L. (2006). Cyclin D1 induction of cellular migration requires p27(KIP1). *Cancer Res.* 66 9986–9994. 17047061 10.1158/0008-5472.CAN-06-1596

[B44] Liang J. Zubovitz J. Petrocelli T. Kotchetkov R. Connor M. K. Han K. (2002). PKB/Akt phosphorylates p27, impairs nuclear import of p27 and opposes p27-mediated G1 arrest. *Nat. Med.* 8 1153–1160. 10.1038/nm761 12244302

[B45] Liebl J. Weitensteiner S. B. Vereb G. Takacs L. Furst R. Vollmar A. M. (2010). Cyclin-dependent kinase 5 regulates endothelial cell migration and angiogenesis. *J. Biol. Chem.* 285 35932–35943. 10.1074/jbc.M110.126177 20826806 PMC2975216

[B46] Liu X. Sun L. Torii M. Rakic P. (2012). Connexin 43 controls the multipolar phase of neuronal migration to the cerebral cortex. *Proc. Natl. Acad. Sci. U.S.A.* 109 8280–8285. 10.1073/pnas.1205880109 22566616 PMC3361458

[B47] Lu Z. Hunter T. (2010). Ubiquitylation and proteasomal degradation of the p21(Cip1), p27(Kip1) and p57(Kip2) CDK inhibitors. *Cell Cycle* 9 2342–2352. 20519948 10.4161/cc.9.12.11988 PMC3319752

[B48] Malek N. P. Sundberg H. McGrew S. Nakayama K. Kyriakides T. R. Roberts J. M. (2001). A mouse knock-in model exposes sequential proteolytic pathways that regulate p27Kip1 in G1 and S phase. *Nature* 413 323–327. 10.1038/35095083 11565035

[B49] McAllister S. S. Becker-Hapak M. Pintucci G. Pagano M. Dowdy S. F. (2003). Novel p27(kip1) C-terminal scatter domain mediates Rac-dependent cell migration independent of cell cycle arrest functions. *Mol. Cell. Biol.* 23 216–228. 10.1128/mcb.23.1.216-228.2003 12482975 PMC140659

[B50] McClellan K. A. Ruzhynsky V. A. Douda D. N. Vanderluit J. L. Ferguson K. L. Chen D. (2007). Unique requirement for Rb/E2F3 in neuronal migration: evidence for cell cycle-independent functions. *Mol. Cell. Biol.* 27 4825–4843. 10.1128/mcb.02100-06 17452454 PMC1951492

[B51] Mitsuhashi T. Aoki Y. Eksioglu Y. Z. Takahashi T. Bhide P. G. Reeves S. A. (2001). Overexpression of p27Kip1 lengthens the G1 phase in a mouse model that targets inducible gene expression to central nervous system progenitor cells. *Proc. Natl. Acad. Sci. U.S.A.* 98 6435–6440. 10.1073/pnas.111051398 11371649 PMC33486

[B52] Montagnoli A. Fiore F. Eytan E. Carrano A. C. Draetta G. F. Hershko A. (1999). Ubiquitination of p27 is regulated by Cdk-dependent phosphorylation and trimeric complex formation. *Genes Dev.* 13 1181–1189. 10.1101/gad.13.9.1181 10323868 PMC316946

[B53] Morelli G. Even A. Gladwyn-Ng I. Le Bail R. Shilian M. Godin J. D. (2018). p27(Kip1) modulates axonal transport by regulating alpha-tubulin acetyltransferase 1 stability. *Cell Rep.* 23 2429–2442. 10.1016/j.celrep.2018.04.083 29791853

[B54] Moriyama K. Iida K. Yahara I. (1996). Phosphorylation of Ser-3 of cofilin regulates its essential function on actin. *Genes Cells* 1 73–86. 10.1046/j.1365-2443.1996.05005.x 9078368

[B55] Moriyama K. Yahara I. (1999). Two activities of cofilin, severing and accelerating directional depolymerization of actin filaments, are affected differentially by mutations around the actin-binding helix. *EMBO J.* 18 6752–6761. 10.1093/emboj/18.23.6752 10581248 PMC1171737

[B56] Nguyen L. Besson A. Heng J. I. Schuurmans C. Teboul L. Parras C. (2006). p27kip1 independently promotes neuronal differentiation and migration in the cerebral cortex. *Genes Dev.* 20 1511–1524. 10.1101/gad.377106 16705040 PMC1475763

[B57] Nishimura Y. V. Nabeshima Y.-I. Kawauchi T. (2017). Morphological and molecular basis of cytoplasmic dilation and swelling in cortical migrating neurons. *Brain Sci.* 7:87. 10.3390/brainsci7070087 28753911 PMC5532600

[B58] Nishimura Y. V. Shikanai M. Hoshino M. Ohshima T. Nabeshima Y. Mizutani K. (2014). Cdk5 and its substrates, Dcx and p27kip1, regulate cytoplasmic dilation formation and nuclear elongation in migrating neurons. *Development* 141 3540–3550. 10.1242/dev.111294 25183872

[B59] Odajima J. Wills Z. P. Ndassa Y. M. Terunuma M. Kretschmannova K. Deeb T. Z. (2011). Cyclin E constrains Cdk5 activity to regulate synaptic plasticity and memory formation. *Dev. Cell* 21 655–668. 10.1016/j.devcel.2011.08.009 21944720 PMC3199337

[B60] Ono K. Nakagawa T. Kojima K. Matsumoto M. Kawauchi T. Hoshino M. (2009). Silencing p27 reverses post-mitotic state of supporting cells in neonatal mouse cochleae. *Mol. Cell. Neurosci.* 42 391–398. 10.1016/j.mcn.2009.08.011 19733668

[B61] Ou L. Ferreira A. M. Otieno S. Xiao L. Bashford D. Kriwacki R. W. (2011). Incomplete folding upon binding mediates Cdk4/cyclin D complex activation by tyrosine phosphorylation of inhibitor p27 protein. *J. Biol. Chem.* 286 30142–30151. 10.1074/jbc.M111.244095 21715330 PMC3191053

[B62] Ovejero-Benito M. C. Frade J. M. (2015). p27(Kip1) participates in the regulation of endoreplication in differentiating chick retinal ganglion cells. *Cell Cycle* 14 2311–2322. 10.1080/15384101.2015.1044175 25946375 PMC4614947

[B63] Phillips A. H. Ou L. Gay A. Besson A. Kriwacki R. W. (2018). Mapping interactions between p27 and RhoA that stimulate cell migration. *J. Mol. Biol.* 430 751–758. 10.1016/j.jmb.2018.01.017 29410088 PMC5965279

[B64] Pippa R. Espinosa L. Gundem G. Garcia-Escudero R. Dominguez A. Orlando S. (2012). p27Kip1 represses transcription by direct interaction with p130/E2F4 at the promoters of target genes. *Oncogene* 31 4207–4220. 10.1038/onc.2011.582 22179826

[B65] Qi G. J. Chen Q. Chen L. J. Shu Y. Bu L. L. Shao X. Y. (2016). Phosphorylation of connexin 43 by Cdk5 modulates neuronal migration during embryonic brain development. *Mol. Neurobiol.* 53 2969–2982. 10.1007/s12035-015-9190-6 25952543

[B66] Roininen N. Takala S. Haapasaari K. M. Jukkola-Vuorinen A. Mattson J. Heikkila P. (2019). Neuroendocrine breast carcinomas share prognostic factors with gastroenteropancreatic neuroendocrine tumors: a putative prognostic role of menin, p27, and SSTR-2A. *Oncology* 96 147–155. 10.1159/000493348 30282082

[B67] Rosen D. G. Yang G. Cai K. Q. Bast R. C. Jr. Gershenson D. M. Silva E. G. (2005). Subcellular localization of p27kip1 expression predicts poor prognosis in human ovarian cancer. *Clin. Cancer Res.* 11 632–637. 15701850

[B68] Russo A. A. Jeffrey P. D. Patten A. K. Massague J. Pavletich N. P. (1996). Crystal structure of the p27Kip1 cyclin-dependent-kinase inhibitor bound to the cyclin A-Cdk2 complex. *Nature* 382 325–331. 8684460 10.1038/382325a0

[B69] Sheaff R. J. Groudine M. Gordon M. Roberts J. M. Clurman B. E. (1997). Cyclin E-CDK2 is a regulator of p27Kip1. *Genes Dev.* 11 1464–1478. 10.1101/gad.11.11.1464 9192873

[B70] Sherr C. J. Roberts J. M. (1999). CDK inhibitors: positive and negative regulators of G1-phase progression. *Genes Dev.* 13 1501–1512. 10.1101/gad.13.12.1501 10385618

[B71] Shikanai M. Nishimura Y. V. Sakurai M. Nabeshima Y. I. Yuzaki M. Kawauchi T. (2018a). Caveolin-1 promotes early neuronal maturation via caveolae-independent trafficking of N-Cadherin and L1. *iScience* 7 53–67. 10.1016/j.isci.2018.08.014 30267686 PMC6135901

[B72] Shikanai M. Yuzaki M. Kawauchi T. (2018b). Rab family small GTPases-mediated regulation of intracellular logistics in neural development. *Histol. Histopathol.* 33 765–771. 10.14670/HH-11-956 29266163

[B73] Shin I. Yakes F. M. Rojo F. Shin N. Y. Bakin A. V. Baselga J. (2002). PKB/Akt mediates cell-cycle progression by phosphorylation of p27(Kip1) at threonine 157 and modulation of its cellular localization. *Nat. Med.* 8 1145–1152. 10.1038/nm759 12244301

[B74] Soppa U. Schumacher J. Florencio Ortiz V. Pasqualon T. Tejedor F. J. Becker W. (2014). The down syndrome-related protein kinase DYRK1A phosphorylates p27(Kip1) and cyclin D1 and induces cell cycle exit and neuronal differentiation. *Cell Cycle* 13 2084–2100. 10.4161/cc.29104 24806449 PMC4111700

[B75] Sutterluty H. Chatelain E. Marti A. Wirbelauer C. Senften M. Muller U. (1999). p45SKP2 promotes p27Kip1 degradation and induces S phase in quiescent cells. *Nat. Cell Biol.* 1 207–214. 10.1038/12027 10559918

[B76] Takahashi Y. Nada S. Mori S. Soma-Nagae T. Oneyama C. Okada M. (2012). The late endosome/lysosome-anchored p18-mTORC1 pathway controls terminal maturation of lysosomes. *Biochem. Biophys. Res. Commun.* 417 1151–1157. 10.1016/j.bbrc.2011.12.082 22227194

[B77] Tanaka H. Yamashita T. Asada M. Mizutani S. Yoshikawa H. Tohyama M. (2002). Cytoplasmic p21(Cip1/WAF1) regulates neurite remodeling by inhibiting Rho-kinase activity. *J. Cell Biol.* 158 321–329. 10.1083/jcb.200202071 12119358 PMC2173114

[B78] Tarui T. Takahashi T. Nowakowski R. S. Hayes N. L. Bhide P. G. Caviness V. S. (2005). Overexpression of p27 Kip 1, probability of cell cycle exit, and laminar destination of neocortical neurons. *Cereb. Cortex* 15 1343–1355. 10.1093/cercor/bhi017 15647527

[B79] Tsvetkov L. M. Yeh K. H. Lee S. J. Sun H. Zhang H. (1999). p27(Kip1) ubiquitination and degradation is regulated by the SCF(Skp2) complex through phosphorylated Thr187 in p27. *Curr. Biol.* 9 661–664. 10375532 10.1016/s0960-9822(99)80290-5

[B80] Viglietto G. Motti M. L. Bruni P. Melillo R. M. D’Alessio A. Califano D. (2002). Cytoplasmic relocalization and inhibition of the cyclin-dependent kinase inhibitor p27(Kip1) by PKB/Akt-mediated phosphorylation in breast cancer. *Nat. Med.* 8 1136–1144. 10.1038/nm762 12244303

[B81] Vlach J. Hennecke S. Amati B. (1997). Phosphorylation-dependent degradation of the cyclin-dependent kinase inhibitor p27. *EMBO J.* 16 5334–5344. 10.1093/emboj/16.17.5334 9311993 PMC1170165

[B82] Yang N. Higuchi O. Ohashi K. Nagata K. Wada A. Kangawa K. (1998). Cofilin phosphorylation by LIM-kinase 1 and its role in Rac-mediated actin reorganization. *Nature* 393 809–812. 10.1038/31735 9655398

[B83] Yang Y. Herrup K. (2007). Cell division in the CNS: protective response or lethal event in post-mitotic neurons? *Biochim. Biophys. Acta* 1772 457–466. 10.1016/j.bbadis.2006.10.002 17158035 PMC2785903

[B84] Zhang J. Li H. Herrup K. (2010). Cdk5 nuclear localization is p27-dependent in nerve cells: implications for cell cycle suppression and caspase-3 activation. *J. Biol. Chem.* 285 14052–14061. 10.1074/jbc.M109.068262 20189989 PMC2859566

